# Effects of introducing a voluntary virtual patient module to a basic life support with an automated external defibrillator course: a randomised trial

**DOI:** 10.1186/1472-6920-12-41

**Published:** 2012-06-18

**Authors:** Andrzej A Kononowicz, Paweł Krawczyk, Grzegorz Cebula, Marta Dembkowska, Edyta Drab, Bartosz Frączek, Aleksandra J Stachoń, Janusz Andres

**Affiliations:** 1 Department of Bioinformatics and Telemedicine, Jagiellonian University Medical College, Lazarza 16, Krakow, 31-530, Poland; 2 Department of Anaesthesiology and Intensive Care Medicine, Jagiellonian University Medical College, Kopernika 17, Krakow, 31-501, Poland

**Keywords:** Virtual patient, BLS-AED training, Blended learning, Voluntary participation

## Abstract

**Background:**

The concept of virtual patients (VPs) encompasses a great variety of predominantly case-based e-learning modules with different complexity and fidelity levels. Methods for effective placement of VPs in the process of medical education are sought. The aim of this study was to determine whether the introduction of a voluntary virtual patients module into a basic life support with an automated external defibrillator (BLS-AED) course improved the knowledge and skills of students taking the course.

**Methods:**

Half of the students were randomly assigned to an experimental group and given voluntary access to a virtual patient module consisting of six cases presenting BLS-AED knowledge and skills. Pre- and post-course knowledge tests and skills assessments were performed, as well as a survey of students' satisfaction with the VP usage. In addition, time spent using the virtual patient system, percentage of screen cards viewed and scores in the formative questions in the VP system throughout the course were traced and recorded.

**Results:**

The study was conducted over a six week period and involved 226 first year medical students. The voluntary module was used by 61 (54%) of the 114 entitled study participants. The group that used VPs demonstrated better results in knowledge acquisition and in some key BLS-AED action skills than the group without access, or those students from the experimental group deliberately not using virtual patients. Most of the students rated the combination of VPs and corresponding teaching events positively.

**Conclusions:**

The overall positive reaction of students and encouraging results in knowledge and skills acquisition suggest that the usage of virtual patients in a BLS-AED course on a voluntary basis is feasible and should be further investigated.

## Background

Virtual patients (VPs) are computer simulations of real-life clinical scenarios created for the purpose of healthcare and medical training, education, or assessment [1]. This concept encompasses a great variety of predominantly case-based e-learning modules with different complexity and fidelity levels [2]. In two recent systematic literature reviews Cook and Triola proposed an agenda for research in the topic of VPs [3,4]. One of the suggested themes was finding effective placement for VPs in the process of medical education. It is vital to know the right balance between computer-based learning (CBL) and non-computer instruction and the type of VP which best suits each group of learners.

Achieving and retaining competency in cardiopulmonary resuscitation is expected from healthcare providers at all levels of education. Methods are sought in order to improve resuscitation training [5]. One possibility is to take advantage of the flexibility offered by e-learning technologies and thus provide students with an additional tool that could be used to prepare and rehearse for traditional face-to-face based classes in their spare time. The introduction of obligatory computer-aided methods at medical schools often meets with resistance in countries with low IT integration in the healthcare system. For that reason a gradual introduction whilst enhancing the curriculum with computer-based methods is preferred. On the other hand, it is possible that voluntary activities may be ignored by students or used unproductively.

In this study the effects of introducing a virtual patients module into an undergraduate course at Jagiellonian University are investigated. The research question is whether the addition of a non-obligatory virtual patient module is worth the effort in terms of improvements in students’ knowledge, skills and satisfaction.

The focus of our study was to examine the utilisation of VPs in the early stages of a medical curriculum to support a traditional (instructor-led) basic life support with automated external defibrillator (BLS-AED) course. A significant amount of evidence regarding the usage of CBL in basic life support (BLS) training is already available [6-10]. None of the studies of computer-aided BLS courses we were aware of focused on undergraduate students of medicine. The studies did not test the effects of the introduction of a web-based VP module on a voluntary basis into an existing curriculum. Hege *et al.* presented their findings from four self-study integration strategies of VPs in medical curricula (but not in BLS courses), these being a mixture of voluntary and obligatory case-based modules presented in addition to lectures and seminars [11]. Their results did not contain any information about knowledge and skills acquisition by students. In a recent study Jensen *et al.* examined the effects of introducing a module of 40 VPs as a learning resource to retain competence acquired from an Advance Life Support course [12]. A lack of social interaction in the e-learning programme was reported as significant barrier to using that module.

The innovation of our study is the broad perspective with which we investigate the outcome of voluntary use of VPs in the BLS-AED training for medical students. Both knowledge and skills improvements after a six week blended learning course are examined, including students’ satisfaction aspects.

## Methods

Before the experiment began students were asked to acknowledge their willingness to participate in the study by signing a consent form. Anonymity of the study participants was ensured by a simple identity encoding system. The study was approved by the ethics committee of Jagiellonian University.

During the course students from the experimental group were granted access to a VP module on the CASUS® platform [13]. The web-based VP shell developed by LMU University of Munich and Instruct AG has frequently been tested in large scale projects and studies in various medical fields [11,14,15]. The CASUS system enables the authoring of VP cases, course management, as well as the tracing of students' activities. All students were given technical instruction on how to use the CASUS platform and practised it several weeks before the study began in the Basics of Computer Science course, working for at least one hour on non-BLS relevant cases.

In order to evaluate the course's educational outcome, all students were tested in BLS-AED knowledge and skills before the course started and after the last meeting of the course. Both tests were unannounced.

The pre- and post-course knowledge tests consisted of 60 true-or-false BLS-AED questions, randomly selected from a pool of 120 questions verified by subject matter experts. The questions were randomised in order to impede the students from memorising them. For each correct answer one point was scored. Both tests were carried out using the Blackboard Academic Suite^TM^ system and were supervised by instructors to prevent students from communicating or using external knowledge sources while taking the test.

The pre- and post-course BLS-AED skills were evaluated using recommendations taken from the standardised Cardiff Test [16]. Each student was given the same scenario: „You are in a shopping mall, suddenly you hear a noise and see a 60-year old woman who collapses in front of you. While falling she knocks a few tins from a shelf”. Additional information for the instructor was: (a) When the student shouts for help a helper immediately appears; (b) The victim is unconscious, not breathing and pulseless when checked; (c) On request AED is available at once. The scenario was terminated after three minutes, including about two minutes BLS time. In cases in which an AED was used the scenario was terminated after two minutes of BLS following first shock delivery. The initial rhythm was always shockable. At the end of their performance students were asked to list criteria for the termination of BLS. Chest compressions and ventilations were performed and recorded on Laerdal Resusci Anne Skill Reporter, on request students received the Powerheart AED G3 Semi Automatic training device by Cardiac Science.

**Figure 1:**
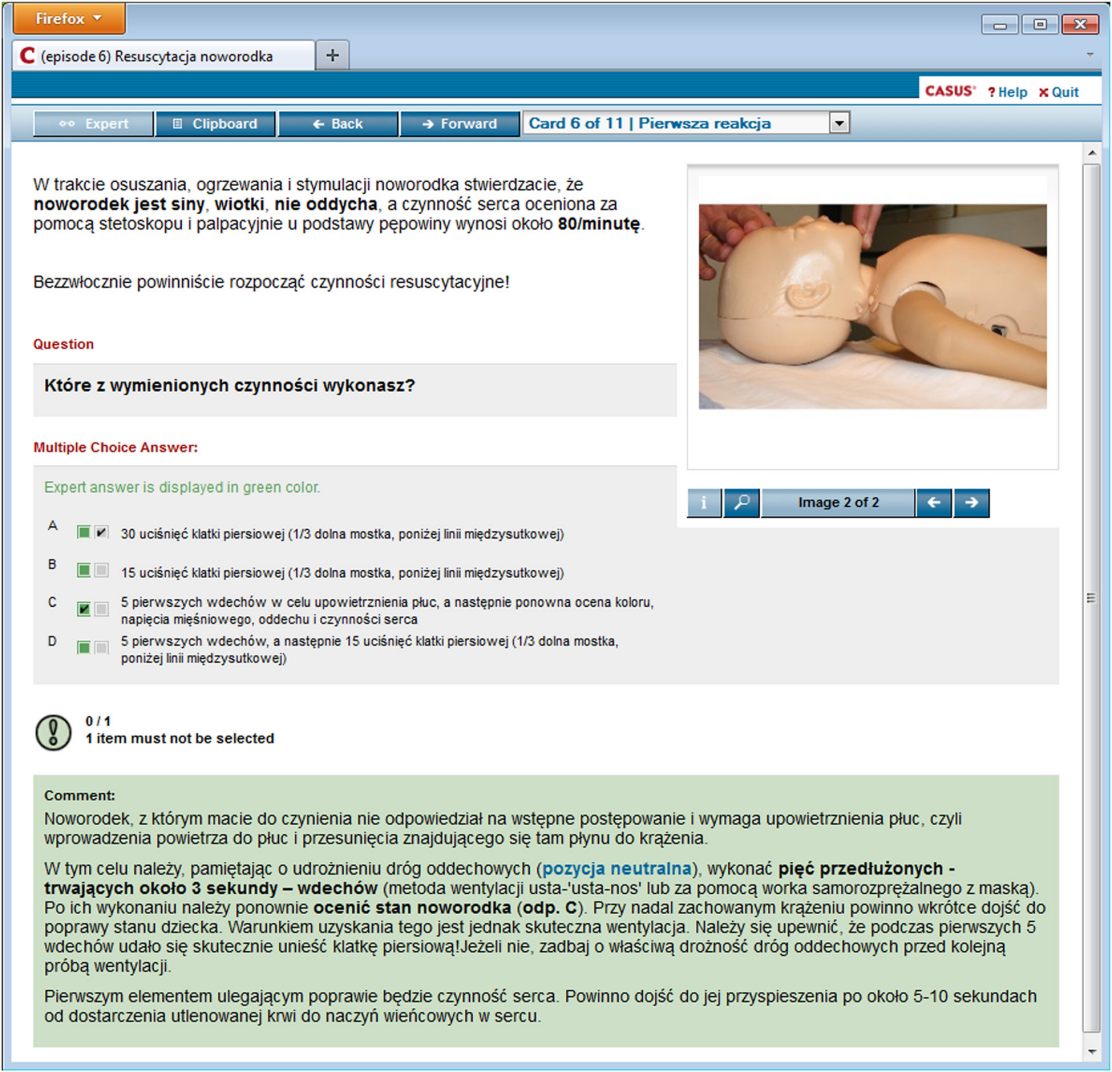
**A sample screen card in the VPs module.** A sample screen card in the VPs module implemented using the CASUS system. A question has been incorrectly answered and feedback with correct answer is displayed. The VP content is in Polish, user interface is selected for English for the purpose of the screenshot – Polish version of the user interface was also available.

Usage of the VP cases was voluntary, but students were encouraged in the lectures to utilise the module to prepare themselves in advance for traditional face-to-face classes. To access the cases students needed to apply for an individual, anonymous account on the platform (no personal data was stored in the system). VPs accompanied a traditional face-to-face instructor-led BLS with AED course consisting of five classes (60 min each). The e-learning module initially contained one single VP case. A new VP case was added to the module every week of the course corresponding to the topics to be taught in the current week. By the end of the course six VPs were available. Table 1 presents the key learning objectives of the cases. All virtual patients were “linear string of pearls” [2] cases consisting of 8 to 17 screen cards with text, images, videos, pop-up windows with additional comments and expert advice, as well as formative questions with feedback. The animations and videos presented key BLS-AED interventions which were also demonstrated in the traditional face-to-face classes. The theoretical foundations of multimedia learning by R. Mayer [17] were followed in the design of the module: text was broken into small segments (screen-cards), essential concepts were highlighted and the text was illustrated by relevant images and animations. Figure 1 presents a sample screen card in the VPs module. It is important to emphasize that the VP module did not contain topics which were not presented in the lectures or the traditional face-to-face classes. Instructors were blinded regarding the study group allocations. In case of technical problems with the VP module usage, students were advised to contact the local CASUS system administrator by e-mail or directly in his office.

**Table 1:** Usage of virtual patient cases by students from the experimental group

**Case**	**Virtual patient**	**Time**	**t/card**	**Score**	**Cmp**
VP1	60-year old male patient loses consciousness in supermarket	17.7	1.77	77%	91%
	**Learning Objectives:** Recognition of the emergency and calling for help. Recovery position.				
VP2	Sudden loss of consciousness of a 67-year old female patient	12.8	1.60	79%	82%
	**Learning Objectives:** Management of the airway. Acute airway obstruction-manual thrusts. Artificial ventilation.				
VP3	50-year old male patient unconscious	15.6	1.73	73%	77%
	**Learning Objectives:** BLS algorithm. Chest compression and ventilation.				
VP4	65-year old male patient loses consciousness in cinema	19.8	1.10	85%	70%
	**Learning Objectives:** Automated External Defibrillation.				
VP5	4-year old girl loses consciousness after choking	14.5	0.91	77%	68%
	**Learning Objectives:** BLS-AED in children.				
VP6	Resuscitation of a newborn	10.3	0.94	52%	62%
	**Learning Objectives:** Resuscitation of newborns.				

Time – Time in minutes spent on average by one student for that virtual patient.

t/card – Time in minutes spent on average by one student on one screen card of the virtual patient.

Score – Average score (in percentage) obtained by one student in that virtual patient.

Cmp – Average percentage of screen cards viewed by a student.

Student satisfaction was measured with questions from a survey proposed by Huwendiek *et al.* to evaluate curricular integration of VPs [18].

Statistical analysis was carried out using the Statsoft Inc., *STATISTICA* 10 package. A significance level *α* of 0.05 was chosen. Knowledge test results in the study groups were compared using ANOVA for repeated measures test. When a significant effect was found, post hoc comparisons with Newman-Keulus test were applied. Dichotomous results of skill assessment were analysed using *χ*^2^ for 3×2 contingency tables. Effect size (ES) was calculated using Cohen’s *d* with pooled standard deviations. Correlation between data was checked with Spearman’s rank correlation coefficient.

All free-text opinions regarding VP integration given by students were carefully read. Using these opinions as its basis, a list of common themes was collated, keywords were assigned, and this was used to index the opinions. Based on the results, less frequent themes (n < 3) were excluded or merged to form more general one.

## Results

### The sample

**Figure 2:**
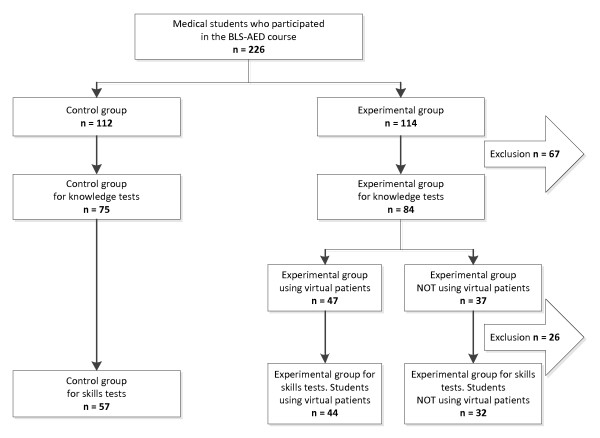
Division of students into study subgroups.

The course was conducted over a six week period up to April 2009 and involved 226 first year medical students (female 138/61%, male 88/39%, median age 19 years) of Jagiellonian University Medical College. It is customary at the university to divide students into groups of 12–15 people in which they usually attend classes throughout their studies. Eight such groups were randomly selected as the experimental group (114 students; female 74/65%; male 40/35%); the remaining eight were the control group (112 students; female 64/57%; male 48/43%). We decided to randomize groups rather than individual students to better separate the experimental and control groups. Figure 2 shows the division of students into study subgroups.

### VP module usage

**Figure 3:**
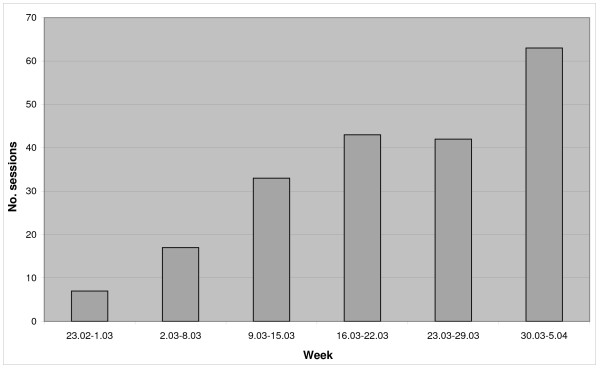
Usage of the VPs module throughout the course.

Sixty one students out of 114 entitled (54%) used the system. The average total time spent on the e-learning module was 91 minutes (SD = 80; Max = 451). On average 15 minutes were spent on each VP case (Table 1). The maximum time spent by one student on one case was 111 minutes. On average students gave 74% correct answers in the formative test questions available in the case. The number of logins (opened user sessions) increased throughout the course (Figure 3). Most of the students used the e-learning system in the evenings with a peak between 9–10 p.m.

### Knowledge acquisition

Out of the 226 students, 67(30%) had to be excluded from the study of knowledge acquisition for not taking the (unannounced) pre- or post-test (n = 61) or due to technical problems (computer breakdown, Internet connection failure, no study identifier given, n = 6 – Figure 2). This formed a sample of 159 students (G_1_: control group, n = 75; G_2_: experimental group deliberately not using VPs, n = 37; G_3_: experimental group using VPs n = 47 – Figure 2).

There was no significant difference in the results of the BLS knowledge pre-test between the experimental and control groups (Table 2). In all groups a significant increase in knowledge between the pre- and post-test was measured (ES_G1_ = 2.27; ES_G2_ = 2.38; ES_G3_ = 3.48; p < 0.001). The variance analysis showed a difference between the groups in terms of the learning outcomes (p = 0.02). Post hoc tests revealed that students’ results in the post-test in the experimental group using virtual patients were significantly higher than in the control group (p < 0.001; ES = 0.73). Comparison of the experimental subgroup using and not using VPs also showed a significant difference (p < 0.001; ES = 0.68). There was no statistically significant difference observed in the post test between the control group and experimental group deliberately not using VPs (p = 0.67).

**Table 2:** Pre- and Post-test results in BLS-AED knowledge

	**G**_**1**_**: n = 75**	**G**_**2**_**: n = 37**	**G**_**3**_**: n = 47**
Pre-Test (mean/SD) [pts]	36.9/4.2	37.4/4.0	36.9/3.4
Post-Test (mean/SD) [pts]	45.8/3.8	46.1/3.4	48.3/3.2

G_1_: Control group, G_2_: Experimental group not using virtual patients, G_3_: Experimental group using virtual patients, SD – Standard deviation, pts – number of points in knowledge test [0–60]. Difference between subgroups is significant according to the ANOVA-test for repeated measurements p = 0.02.

No correlation was found between the total usage time of the VP module and post-test score (Spearman R = 0.17; p = 0.26), nor between the percentage of correct answers in the formative assessment in CASUS and the post-test score (Spearman R = 0.03; p = 0.83). However, a correlation between the percentage of screen cards viewed by students (completion rate) and their post-test score could be demonstrated (Spearman R = 0.33; p = 0.03).

### Skill acquisition

From the group which was available for the knowledge acquisition study, 26 students had to be excluded at the stage of skills acquisition test due to not showing up at the skills assessment (n = 13), refusal to demonstrate their skills on a manikin (n = 7) or technical problems with the printer in the recording device (n = 6) – Figure 2.

There were no statistically significant differences between the groups in the pre-test of skills. The experimental group scored better in some post-course skills assessment than the control group (Table 3). Statistically significant changes were observed while opening airways (G_1_:70.2%/G_2_:78.1%/G_3_:97.7%; p < 0.001), checking for signs of circulation (G_1_:59.7%/G_2_:71.9%/G_3_:88.6%; p = 0.004) and knowledge of conditions in which to stop BLS (G_1_:14.0%/G_2_:21.9%/G_3_:30.26%; p = 0.03).

**Table 3:** Post-test results in BLS-AED skills

	**G**_**1**_**: n = 75**	**G**_**2**_**: n = 37**	**G**_**3**_**: n = 47**	**p**
	**%**	**%**	**%**	
Safe approach performed	73.7%	78.1%	84.1%	0.45
Arouse shout performed	91.2%	96.9%	100.0%	-
Shake performed correctly	87.7%	90.6%	100.0%	-
Shout for help performed	75.4%	68.8%	70.5%	0.76
Open airway performed correctly	70.2%	78.1%	97.7%	**<0.001**
Check for signs of circulation correctly	59.7%	71.9%	88.6%	**0.004**
Phone for help performed	87.7%	96.9%	97.7%	-
Shout for AED	29.8%	28.1%	27.3%	0.96
Start with chest compression	93.0%	96.9%	93.2%	-
Correct identification of place for chest compression	93.0%	93.8%	95.5%	-
Correct C:V Ratio (30:2)	98.3%	100.0%	95.5%	-
Knows when to stop BLS	14.0%	21.9%	36.4%	**0.03**
No unnecessary breaks during BLS	82.4%	78.1%	86.4%	0.64
Average ventilation volume in [500–1000] ml	64.9%	68.8%	68.2%	0.91
Average compression depth in [40–50] mm	42.1%	34.4%	40.9%	0.76
If AED requested	**n = 17**	**n = 9**	**n = 12**	
Safe use of AED:	81.3%	33.3%	81.8%	-
Correct electrode pad placement	68.8%	66.7%	100.0%	-

G_1_: Control group, G_2_: Experimental group not using virtual patients, G_3_: Experimental group using virtual patients, p value in the *χ*^2^ test for 3×2 contingency table, reported if test assumptions were met.

### Students’ satisfaction

All 47 students from the experimental group who used VPs filled in an evaluation questionnaire created by Huwendiek *et al.*[18]. The results of this survey are presented in Table 4. Most of the students positively rated the combination of VPs and corresponding teaching events as a worthwhile learning experience (mean = 4.5; SD = 0.5). The instructors of the students who used VPs were well prepared for the corresponding teaching event (mean = 4.4; SD = 0.7), and the chronological order of the virtual patient work and the corresponding teaching events was well thought out (mean = 4.3; SD = 0.7). Contrastingly, the instructor-led classes are still regarded by most students as indispensable, which can be clearly seen from the fact that most of the students agreed with the statement that the corresponding teaching events gave them an insightful learning experience which they would not have had from VPs alone (mean = 4.6; SD = 0.6). The corresponding teaching activities gave the students a positive climate for learning (mean = 4.6; SD = 0.5) and the feeling of being part of a ‘community’ (mean = 4.3; SD = 0.7). Most of the students felt well informed about how VPs were integrated into the BLS course (mean = 4.4; SD = 0.6) and had easy access to the e-learning module (mean = 4.5; SD = 0.8). However, the evaluation of open-ended questions showed that there were still students for whom computers and Internet access formed a significant barrier.

**Table 4:** Students’ satisfaction with curricular integration of VPs

**id**	**Question**	**G**_**3**_**: n = 47**
		**mean/SD**
1.	I felt well-informed about how the virtual patients were integrated into this course	4.4/0.6
2.	The chronological order of the virtual patient work and the corresponding teaching events was well thought out.	4.3/0.7
3.	The time spent on the virtual patients was well balanced with the time spent on the corresponding teaching events.	4.2/0.8
4.	The content of virtual patients and the corresponding teaching events complemented each other well	4.1/0.8
5.	The corresponding teaching events gave me an insightful learning experience, which I would not have had from the virtual patients alone.	4.6/0.6
6.	I think that learning with the virtual patients is important in order to do well in the final exam for this course	4.0/0.7
7.	I had easy access to the virtual patients at my convenience.	4.5/0.8
8.	The teachers helped me to assess my learning during the corresponding teaching events	3.9/0.7
9.	The teachers facilitated the further development of my clinical reasoning skills during the corresponding teaching events	4.2/0.7
10.	The teachers were well prepared for the corresponding teaching events (incl. familiarity with the virtual patients).	4.4/0.7
11.	I was actively involved in critically weighing pros and cons for explanations given by other students during the corresponding teaching events.	3.6/0.9
12.	I was actively involved in applying my newly gained insights in clinical reasoning, during the corresponding teaching events	4.1/0.7
13.	I was actively involved in refining my clinical reasoning skills during the corresponding teaching events.	4.1/0.7
14.	The quality of discussion during the corresponding teaching events was good.	4.2/0.7
15.	I felt secure enough to openly discuss even my shortcomings (*e.g.* my mistakes while working with virtual patients) during the corresponding teaching events.	4.1/0.7
16.	I felt a positive climate for learning during the corresponding teaching events	4.6/0.5
17.	I felt like part of a ‘community’ during the corresponding teaching events.	4.3/0.7
18.	The combination of virtual patients and corresponding teaching events enhanced my clinical reasoning skills	4.1/0.8
19.	The combination of virtual patients and corresponding teaching events made me feel better prepared to care for a real life patient with this complaint.	4.0/0.7
20.	Overall, the combination of virtual patients and corresponding teaching events was a worthwhile learning experience.	4.5/0.5

G_3_: Experimental group using virtual patients, SD – Standard Deviation. Reported scores are in Likert scale: 1. Strongly disagree, 2. Disagree, 3. Neither agree nor disagree, 4. Agree, 5. Strongly agree.

Open-ended questions in the questionnaire formed an opportunity for the students to elaborate on their personal opinion on VPs themselves, as well as their integration into the course. About half of the students who used VPs stated in their free text opinions that the acquisition of knowledge from the e-learning module was easier for them than from books, and gave them new insights on problems discussed in traditional classes (n = 24). On the other hand, these opinions were partially balanced by other students stating their dislike of learning on-line (n = 4) or the overly complex login process (n = 11). One student stated that VPs were a good opportunity to prepare well for the final exams while instructor-led classes with manikins gave them more confidence to apply the knowledge in practice. Students identified as challenges facing VPs implementation: lack of confidence in VP efficacy expressed by some instructors from the faculty (n = 16), lack of time (n = 14), not enough information about the possibility of voluntary access of VPs (n = 13), the lack of good Internet access (n = 8) and the lack of on-line feedback given directly by teachers (n = 3). Surprisingly, some students criticised the fact that usage of the VP module was voluntary (n = 6), which had a negative impact on their motivation to use it. Among the advantages of the introduction of the VP module noticed by students were: flexibility of time and place of learning (n = 16), exam-relevance (n = 11), the possibility for self-assessment or to learn from errors (n = 11), practical usefulness (n = 10), belief in higher efficiency of this learning method in comparison to traditional learning methods (n = 6), multimedia content and the interactivity of the assignments (n = 5), the motivation to learn regularly (n = 3). One of the students summarised the VPs module advantages as: "you do not have to copy it, you cannot lose it, and it takes you just 10 minutes weekly".

## Discussion

The study was conducted in an actual university setting which shows students’ motivation to use a VP module better than it is possible in a more rigidly controlled artificial environment. It is definitely encouraging to notice that students from the intervention group using virtual patients were able to gain more from the course and scored better in the knowledge post-test than the control group. The voluntary participation percentage was much higher than that reported in voluntary courses by Hege at al. [11] but we have not investigated the reasons for this fact which could be the subject of a future study. A positive aspect of the instructor-led classes was that they gave the students the desired "social interaction" which is often missing in e-learning activities [12,19,20]. Worth stressing is the fact that the percentage of students correctly recognising a cardiac arrest victim was significantly higher in the experimental group than in the other groups. The lack of significant correlation between the amount of intervention and the score in the post-test can be explained by the fact that time spent on a screen card is only a very rough indicator of the thoroughness of a case session, and can be influenced by external factors like, for instance, learning in parallel from a text book, the presence of learning distractors or a slow Internet connection [11]. Nevertheless, the demonstrated correlation between the percentage of screen cards viewed by students and their post-test score is again a very positive sign.

Based on our experience collected in the described implementation we would like to suggest that educators who plan to introduce new projects including virtual patients in the existing curriculum should focus on stimulation of students’ motivation to participate in the project. The first action appears to be initiation of the course with a dedicated pilot learning activity to show the students how to use the new software efficiently. In our case, this activity was combined with “basics of computer science” classes and was appreciated by course participants. Similar experiences can be found in recent literature [21,22]. The second action concerns finding a way to make teachers more enthusiastic about promoting the additional learning activity to the students. The main challenge might be to fight the resistance and inertia towards new technology among faculty members, which in some cases could even be called technophobia [19]. We believe that good integration of the VPs episodes with practical part of the course has the potential to increase motivation and strengthen the course continuum. As suggested by Hege *et al.*[11], the exam relevance of the content also seems to be a good stimulus.

Some students indicated problems accessing VPs because of Internet connection or computer access limitations. The solution for that would be to enable the VP application to be displayed on mobile devices.

We also suggest making the login process as simple as possible. In our study the complex login procedure was caused by the need for anonymous identification of each student in order to compare pre- and post-test results with students activity in the VP system. However, this was recognised by students as significant barrier in the VP access. Despite the fact that every student should have received an e-mail about VPs, some of them complained about a lack of information about VP introduction. We suspect this could be caused by the rarely checked e-mail accounts provided in the project’s initiation phase, or by spam filters which were too sensitive. We were not using students' official university e-mail accounts since they were not often checked by students.

Beyond the scope of the study, but worth noticing, was data describing the quality of BLS-AED skills education in general. During post-course skills assessment only one-third of students asked for AED when faced with an unconscious, non-breathing person, which is an obvious improvement compared to the pre-course assessment in which nobody requested AED, however, 30% is still a very poor result. Also the BLS skills of students after the course did not in many cases meet the criteria concerning the correct depth of compressions and the quality of ventilation as defined by 2005 Resuscitation Guidelines [23]. Similar poor results have also been obtained in other recent studies [6,9]. This issue is currently undergoing further investigation.

### Study limitations

One of the limitations of the study was surely the fact that a relatively large part of the experimental group (46%) did not use the e-learning module influencing the study design. This is, however, the inevitable consequence of the selected research question dealing with the voluntary use of VPs. It was outside the control of the authors to determine this percentage in advance to make the study subgroups more even in terms of the number of participants.

A major limitation with this sort of study that should be acknowledged, is that the students who accessed the VP resources were able to spend more time learning the subject, thus it is no surprise that they did better in the knowledge test. On the other hand, we have also no proof that students from the control group and the experimental group not using virtual patients did not study the subject in their spare time using methods other than virtual patients (*e.g.* information found on the Internet).

The observed effect size between the control group and the experimental group using virtual patients is 0.73. It needs to be recognised as a limitation of this study that this result may be affected by the volunteer bias effect since volunteers are usually more motivated to learn than other students [24]. On the other hand, a comparison between the control group which surely included students willing to learn more in their free time and the experimental subgroup deliberately not using virtual patients showed no difference (45.8/46.1, p = 0.67). The control group was inferior to the merged experimental group containing both volunteers and those students who were not willing to use virtual patients (45.8/47.4, p = 0.01 in contrast analysis) but with an effect size of ES = 0.44.

Even though attempts were made to lessen interaction between control and experimental group members by randomization based on the students groups rather than individual students, their mutual influence cannot be excluded in the selected study design. This might have blurred the observed differences between the groups. The relatively high number of study drop-outs can be explained by the fact that the pre- and post-tests were unannounced and that the selected identity encoding method (based on selected letters from personal data known only by the students) was sometimes confusing for the study participants. When there was any doubt we preferred to exclude the participant from the study rather than to risk data inconsistency.

## Conclusions

The overall positive response of students and encouraging results in knowledge and skills acquisition suggest that the usage of VP cases on a voluntary basis in a BLS-AED is feasible. The participants of the presented course clearly benefited from the additional module and the students approved this innovation and used it effectively. We anticipate an increase in coming years in the percentage of students able and eager to learn this way. A study is planned to analyse the factors which make students willing to participate in voluntary educational scenarios involving VPs and methods of increasing motivation to use VPs.

## Competing interests

The authors declared that they have no competing interest.

## Authors' contributions

AK and PK conceived and designed the study as well as drafted the first version of the manuscript. PK was responsible for the medical content of virtual patients presented in the course. AK, PK and AS were responsible for the technical aspects of the course and authoring of new multimedia content. PK, GC, MD, ED, BF collected data, participated in the choice of knowledge and skills measures, evaluated students’ interventions, were involved as teachers in the BLS-AED course and participated in the interpretation of data. AK performed the statistical data analysis. AS made substantial contributions to the interpretation of data. JA coordinated the project and critically revised the paper. All authors read and approved the final manuscript.

## Pre-publication history

The pre-publication history for this paper can be accessed here:

http://www.biomedcentral.com/1472-6920/12/41/prepub

## References

[B1] Ellaway R Poulton T Fors U McGee JB Albright S Building a virtual patient commons Med Teach 2008 30 170 174 10.1080/01421590701874074 18464142

[B2] Huwendiek S de Leng BA Zary N Fischer MR Ruiz JG Ellaway R Towards a typology of virtual patients Med Teach 2009 31 743 748 10.1080/01421590903124708 19811212

[B3] Cook DA Triola MM Virtual patients: a critical literature review and proposed next steps Med Educ 2009 43 303 311 10.1111/j.1365-2923.2008.03286.x 19335571

[B4] Cook DA Erwin PJ Triola MM Computerized virtual patients in health professions education: a systematic review and meta-analysis Acad Med 2010 85 1589 1602 10.1097/ACM.0b013e3181edfe13 20703150

[B5] Perkins GD Mancini ME Resuscitation training for healthcare workers Resuscitation 2009 80 841 842 10.1016/j.resuscitation.2009.06.013 19573976

[B6] Monsieurs KG Vogels C Bossaert LL Meert P Manganas A Tsiknakis M Leisch E Calle PA Giorgini F Learning effect of a novel interactive basic life support CD: the JUST system Resuscitation 2004 62 159 165 10.1016/j.resuscitation.2004.02.014 15294401

[B7] Moule P Evaluation of the Basic Life Support CD-ROM: Its effectiveness and learning tool and user experiences Educ Technol Soc 2002 5 163 174

[B8] Moule P Albarran JW Bessant E Brownfield C Pollock J A non-randomized comparison of e-learning and classroom delivery of basic life support with automated external defibrillator use: a pilot study Int J Nurs Pract 2008 14 427 434 10.1111/j.1440-172X.2008.00716.x 19126070

[B9] Reder S Cummings P Quan L Comparison of three instructional methods for teaching cardiopulmonary resuscitation and use of an automatic external defibrillator to high school students Resuscitation 2006 69 443 453 10.1016/j.resuscitation.2005.08.020 16678958

[B10] de Vries W Handley AJ A web-based micro-simulation program for self-learning BLS skills and the use of an AED. Can laypeople train themselves without a manikin? Resuscitation 2007 75 491 498 10.1016/j.resuscitation.2007.05.014 17629390

[B11] Hege I Kopp V Adler M Radon K Mäsch G Lyon H Fischer MR Experiences with different integration strategies of case-based e-learning Med Teach 2007 29 791 797 10.1080/01421590701589193 18236274

[B12] Jensen ML Mondrup F Lippert F Ringsted C Using e-learning for maintenance of ALS competence Resuscitation 2009 80 903 908 10.1016/j.resuscitation.2009.06.005 19570601

[B13] Fischer MR CASUS – An authoring and learning tool supporting diagnostic reasoning ZFHD 2000 1 87 98

[B14] Fall LH Berman NB Smith S White CB Woodhead JC Olson AL Multi-institutional development and utilization of a computer-assisted learning program for the pediatrics clerkship: the CLIPP Project Acad Med 2005 80 847 855 10.1097/00001888-200509000-00012 16123465

[B15] Stark R Kopp V Fischer MR Case-based learning with worked examples in complex domains: Two experimental studies in undergraduate medical education Learn Instr 2011 21 22 33 10.1016/j.learninstruc.2009.10.001

[B16] Whitfield RH Newcombe RG Woollard M Reliability of the Cardiff Test of basic life support and automated external defibrillation version 3.1 Resuscitation 2003 59 291 314 10.1016/S0300-9572(03)00246-6 14659599

[B17] Mayer RE Applying the science of learning to medical education Med Educ 2010 44 543 549 10.1111/j.1365-2923.2010.03624.x 20604850

[B18] Huwendiek S Haider HR Tönshoff B de Leng BA Evaluation of curricula integration of virtual patients: Development of a student questionnaire and a reviewer checklist within the Electronic Virtual Patient (eViP) project Bio-Algorithms and Med-Systems 2009 5 35 44

[B19] Childs S Blenkinsopp E Hall A Walton G Effective e-learning for health professionals and students–barriers and their solutions. A systematic review of the literature–findings from the HeXL project Health Info Libr J 2005 22 Suppl 2 20 32 16279973 10.1111/j.1470-3327.2005.00614.x

[B20] Cook DA Web-based learning: pros, cons and controversies Clin Med 2007 7 37 42 17348573 10.7861/clinmedicine.7-1-37 PMC4953546

[B21] Hull P Chaudry A Prasthofer A Pattison G Optimal sequencing of bedside teaching and computer-based learning: a randomised trial Med Educ 2009 43 108 112 10.1111/j.1365-2923.2008.03261.x 19161479

[B22] Tsai CW Involving students in a blended course via teacher's initiation in Web-enhanced collaborative learning Cyberpsychol Behav Soc Netw 2010 13 577 580 10.1089/cyber.2009.0287 20950184

[B23] Handley AJ Koster R Monsieurs K Perkins GD Davies S Bossaert L European Resuscitation Council guidelines for resuscitation 2005. Section 2. Adult basic life support and use of automated external defibrillators Resuscitation 2005 67 Suppl 1 s7 s23 16321717 10.1016/j.resuscitation.2005.10.007

[B24] Callahan CA Hojat M Gonnella JS Volunteer bias in medical education research: an empirical study of over three decades of longitudinal data Med Educ 2007 41 746 753 10.1111/j.1365-2923.2007.02803.x 17661882

